# HIV behind Bars: Human Immunodeficiency Virus Cluster Analysis and Drug Resistance in a Reference Correctional Unit from Southern Brazil

**DOI:** 10.1371/journal.pone.0069033

**Published:** 2013-07-09

**Authors:** Isabel M. Prellwitz, Brunna M. Alves, Maria Letícia R. Ikeda, Daniele Kuhleis, Pedro D. Picon, Carla A. Jarczewski, Marta R. Osório, Alexandra Sánchez, Héctor N. Seuánez, Bernard Larouzé, Marcelo A. Soares, Esmeralda A. Soares

**Affiliations:** 1 Programa de Genética, Instituto Nacional de Câncer, Rio de Janeiro, Brazil; 2 Secretaria de Saúde de Viamão, Prefeitura de Viamão, Viamão, Brazil; 3 Departamento de Ensino e Pesquisa, Hospital Sanatório Partenon, Porto Alegre, Brazil; 4 Secretaria de Segurança Pública, Governo do Estado do Rio Grande do Sul, Porto Alegre, Brazil; 5 Fundação Estadual de Produção e Pesquisa em Saúde, Governo do Estado do Rio Grande do Sul, Porto Alegre, Brazil; 6 Escola Nacional de Saúde Pública, Fundação Oswaldo Cruz, Rio de Janeiro, Brazil; 7 Secretaria de Estado de Administração Penitenciária, Governo do Estado do Rio de Janeiro, Rio de Janeiro, Brazil; 8 Departamento de Genética, Universidade Federal do Rio de Janeiro, Rio de Janeiro, Brazil; 9 INSERM U707, Paris, France; 10 Université Pierre et Marie Curie - Paris 6, Paris, France; Institut Pasteur, France

## Abstract

People deprived of liberty in prisons are at higher risk of infection by the human immunodeficiency virus (HIV) due to their increased exposure through intravenous drug use, unprotected sexual activity, tattooing in prison and blood exposure in fights and rebellions. Yet, the contribution of intramural HIV transmission to the epidemic is scarcely known, especially in low- and middle-income settings. In this study, we surveyed 1,667 inmates incarcerated at Presídio Central de Porto Alegre, located in southern Brazil, for HIV infection and molecular characterization. The HIV seroprevalence was 6.6% (110/1,667). Further analyses were carried out on 40 HIV-seropositive inmates to assess HIV transmission clusters and drug resistance within the facility with the use of molecular and phylogenetic techniques. The molecular epidemiology of HIV-1 subtypes observed was similar to the one reported for the general population in southern Brazil, with the predominance of HIV-1 subtypes C, B, CRF31_BC and unique BC recombinants. In particular, the high rate (24%) of URF_BC found here may reflect multiple exposures of the population investigated to HIV infection. We failed to find HIV-infected inmates sharing transmission clusters with each other. Importantly, the analysis of HIV-1 *pol* genomic fragments evidenced high rates of HIV primary and secondary (acquired) drug resistance and an alarming proportion of virologic failure among patients under treatment, unveiling suboptimal access to antiretroviral therapy (ARV), low ARV adherence and dissemination of drug resistant HIV strains in primary infections. Our results call for immediate actions of public authority to implement preventive measures, serological screening and, for HIV-seropositive subjects, clinical and treatment follow-up in order to control HIV infection and limit the spread of drug resistance strains in Brazilian prisons.

## Introduction

Despite HIV/AIDS is a pandemic with worldwide and cross-strata proportions, HIV commonly disseminates more rapidly in specific groups, such as those of men who have sex with men (MSM), commercial sex workers, intravenous drug users (IDU) and people deprived of liberty [1]–[4]. In prisons, HIV prevalence rates are frequently several-fold higher when compared to the general population of the same locale, especially in low- and middle-income countries [5]. Incarcerated subjects are frequently exposed to conditions that favor HIV dissemination, such as IDU, unprotected sexual activity, promiscuity, tattooing and exposure to contaminated blood during fights and rebellions [6]. Moreover, inmates are at increased risk of exposure to co-infections with other viral (e.g., hepatitis B and C) and bacterial (e.g., tuberculosis, syphilis) agents that greatly increase susceptibility to HIV infection [5].

In Brazil, a middle-income country with continental dimensions, over 600,000 HIV-infected persons are estimated as of June 2011. The South region of the country is of special concern, being the area with the highest reported AIDS incidence in the last years [7]. There are estimated 514,582 incarcerated subjects in Brazil, while the South region concentrates almost 20% of this population (http://www.infopen.gov.br/). Despite such high numbers of prisoners, scarce information is known about their HIV/AIDS status and associated conditions. In particular, the contribution of HIV transmission during incarceration is largely unknown in the Brazilian penitentiary system, with few studies addressing this issue [8], [9].

HIV-1M (*major*), the pandemic variant of the AIDS virus, is classified into nine pure subtypes (A-D, F-H, J, K) and numerous circulating intersubtype recombinant forms (CRFs) [10]. While in Brazil HIV-1 subtype B is the prevailing strain, the south of the country is unique in the Brazilian epidemic, with an endemic focus of HIV-1 subtype C, a variant of African origin that has disseminated in the area in the last three decades [11]–[17]. This unique focus of HIV-1C in the Americas has created an interesting scenario for HIV-1 molecular epidemiology studies in the area.

In view of the abovementioned scenario, the present study aims to characterize the impact of intraprison HIV transmission and of HIV drug resistance to the HIV/AIDS epidemic of a large penitentiary unit in southern Brazil through the use of molecular identification techniques. HIV-1 subtypes, as well as CRFs and unique recombinant forms (URFs) have been defined in recently-diagnosed, HIV-infected inmates identified through a serological survey and transmission clusters and drug resistance strains have been assessed.

## Materials and Methods

### Subjects

This cross sectional study was conducted as part of a larger project financed by the Global Fund on Tuberculosis/Brazil. Between September and November 2009, 1,667 sentenced inmates incarcerated at the aisles B and C of the Presídio Central de Porto Alegre (n = 4,700), a reference Brazilian correctional system center located in southern Brazil, were screened for TB and HIV using commercial-based rapid tests following the Brazilian Ministry of Health guidelines. The screened subjects represented approximately 90% of the inmates incarcerated in these two aisles and 40% of the inmates in the prison. Of the 1,667 inmates tested for HIV, one hundred and ten (6.6%) tested positive for HIV-1. Four months later, these 110 inmates were looked for to participate in this survey, but 70 of them had already been transferred to another prison or liberated, and only 40 were still in the prison. A five milliliter sample was further collected from these 40 subjects specifically for the present study. Each enrolled inmate was individually explained the objectives of the study and agreed to participate in it and to sign a written informed consent. No subjects declined to participate in the study. Together with the blood sample, a questionnaire was applied to each inmate to collect socio-demographic (age, time of incarceration), behavioral (illicit drug use, sexual activity within and outside the prison, use of condoms, tattooing within the prison) and clinical (previous/current sexually transmitted diseases and other clinical events, HIV-1 viral load, CD4^+^ T-cell counts, antiretroviral treatment status, treatment history when applicable) data. Questionnaire data was compiled in Microsoft Excel for further analyses. This study and the written consent form were approved by the Ethics Committee of the RS State Foundation for Scientific Production and Research (FEPPS-RS), RS State Secretary of Health, and by the Brazilian National Committee on Ethics in Research (CONEP).

### HIV Molecular Analyses

Blood samples from HIV-seropositive inmates were sent to the Brazilian Cancer Institute (INCA) at Rio de Janeiro for further HIV molecular analysis. Plasma was separated by centrifugation, and the buffy coat was used for genomic DNA extraction with the QIAamp DNA Mini Kit (QIAGEN, Chatsworth, USA), following manufacturer’s specifications. Nested PCR reactions were carried out for the amplification of two HIV genomic fragments of approximately 900 bp each. The first fragment covered the viral protease (PR) and the reverse transcriptase (RT) polymerase domain (the first 225 RT codons), while the second covered the RT C-terminal regions (connection - CN and RNase H - RNH). These fragments were amplified with PCR primers and conditions previously described [18], [19]. In cases where the fragments could not be successfully obtained, the four HIV-1 *pol* regions (PR, RT-pol, RT-CN and RT-RNH were amplified separately as previously described [18]–[21]. Amplified PCR products were visualized in 1% agarose gels and purified with the Illustra GFX PCR DNA Purification kit (GE Healthcare, Piscataway, USA), quantified and labeled with Big Dye Terminator kit (Life Technologies, Carlsbad, USA) with the same primers used in the second round PCR. Labeled samples were run in an automated 3130XL Genetic Analyzer (Life Technologies) and sequences were edited and assembled with SeqMan (DNAStar, Madison, USA). HIV-1 sequences suggestive of mixed multiple variants had their PCR products further subject to DNA cloning into pMOS Blue Blunt-Ended plasmid (GE Healthcare) following manufacturer’s instructions. Recombinant plasmids were transformed into *E.coli* DH5α chemically competent cells (Life Technologies). Colonies were screened for the presence of the appropriate insert by colony PCR with M13 universal primers, and positive clones were sequenced and analyzed as described above. An average of 19 (8–53) clonal sequences was analyzed from each sample.

All HIV-1 sequences generated herein have been submitted to the GenBank database and were assigned the accession numbers KC286150 to KC286485.

### Phylogenetic and Recombination Analyses

HIV-1 sequences in FASTA format were aligned with HIV-1 subtype reference sequences retrieved from the HIV Los Alamos Database (http://hiv-web.lanl.gov) using BioEdit Sequence Alignment Editor v7.0.9.0 [22]. HIV-1 subtype classification of each query sequence was inferred through phylogenetic analysis by clustering with specific HIV subtype reference sequences. PhyML v.3.0 [23] was used for maximum likelihood (ML) analysis using the best model of nucleotide substitution inferred with Model Generator [24]. Clade robustness was evaluated with the approximate likelihood ratio test (aLRT) [25]. The trees generated were visualized using FigTree v.1.3.1 (publicly available at http://tree.bio.ed.ac.uk/). Phylogenetic analyses were also performed using the neighbor-joining (NJ) method and Kimura’s two-parameter correction with MEGA 5.0 [26] and 1,000 bootstrap replicates. Sequences not grouped with any HIV-1 subtype/CRF were subsequently analyzed with the *bootscanning* tool of Simplot v.3.5.1 [27] for determining patterns of recombination and the HIV-1 subtypes involved in the recombination event. The following parameters were used: window = 200 pb; steps = 20 pb; T/t = 2.0; gapstrip = on; replicas = 100; Kimura 2-parameter; Neighbor-Joining. Recombinant strains were further confirmed by phylogenetic analysis of individual HIV-1 subtype genomic fragments as suggested by the *bootscanning* breakpoint analysis (data not shown).

### Transmission Cluster Analysis

For the identification of potential intraprison transmission clusters, phylogenetic analyses were carried out as described above with all viral sequences generated herein (either uncloned or clonal). Sequences were first grouped according to their HIV-1 subtype/recombinant assignment and each group was aligned with additional HIV sequences from unrelated subjects attending a public hospital in the city of Porto Alegre (local controls - LC) and with best matched HIV-1 sequences retrieved in BLAST searches with the putative clustered sequences. Inmate HIV sequences were considered from the same cluster if they grouped together with high aLRT or bootstrap support without any intervenient LC or BLAST sequence.

### Antiretroviral Drug Resistance Analysis

Viral *pol* sequences were analyzed for the presence of drug resistance mutations through genotypic interpretation. Protease and RT-pol sequences were subject to the Stanford HIV Drug Resistance algorithm available online at http://hivdb.stanford.edu/. Drug resistance mutations were analyzed based on the consensus of the International AIDS Society [28]. For the RT-CN and -RNH regions, a manual inspection analysis was carried out and included the following mutations and polymorphisms recently described and confirmed to impact drug resistance: G335D/C, N348I, A360V, T369I/V, A371V, A376S, A400T in CN; D488E, Q509L and Q547K in RNH [18], [29]–[35].

## Results

One hundred ten inmates were diagnosed as HIV-infected in a screening of 1,667 subjects at PCPA, representing a total prevalence of 6.6%. Of those, a second blood sample from 40 (36%) individuals was available for analysis, and for 38 of the latter (95%) molecular analysis yielded viral sequence information.

Table 1 summarizes available socio-demographic and clinical characteristics of the 38 subjects. Median age was 31.5 years (SD = 7.1) and median time of imprisonment was 38 months (SD = 37.6). Thirty seven (97%) had reported drug use, including intravenous cocaine use, cocaine inhalation, crack or marijuana. Ten subjects (26%) reported sexual encounters with commercial sex workers either previous to or during incarceration, and 14 (37%) had tattooed in prison.

**Table 1:** Demographic, behavioral and clinical characteristics of HIV-infected inmates (n = 38).

**Median age (years)**	31.5 (SD* 7.14)	
**Median time of incarceration (months)**	38 (SD 37.6)	
**Risk behavior**		
Drug use	37 (97%)	
Relations with sex workers beforeincarceration	10 (26%)	
Tattooing in prison	14 (37%)	
Sexually Transmitted Disease (STD)before or during incarceration	18 (47%)	
Do not use/occasional use of condomswithin the prison	04 (11%)	
Known HIV^+^ status at study entry	14 (37%)	
**Clinical characteristics**		
Previous Tuberculosis	17 (44%)	
Antiretroviral treatment experience		
Treated	7 (18%)	
Untreated	31 (82%)	
Median HIV viral load (copies/ml)		
Treated	26,598 (SD 51,862)	
Untreated	10,848 (SD 59,816)	
Total	11,409 (SD 59,816)	
Undetectable HIV viral load**		
Treated	2	
Untreated	1	
Median CD4^+^ T-cell counts (cells/mm^3^)		
Treated	269 (SD 210,3)	
Untreated	424 (SD 292,2)	
Total	391 (SD 288)	

* SD – standard deviation.

** <80 copies/ml plasma.

Seventeen (44%) inmates had previous tuberculosis (TB) infection in the last 12 months (all resolved at the time of sample collection), and 18 (47%) had history of previous sexually transmitted diseases (STD). Fourteen subjects (37%) had previous knowledge of their HIV^+^ status, and seven (18%) had reported previous use of antiretroviral therapy. Median CD4^+^ T-cell counts among previously treated inmates were 269 cells/mm^3^ of blood, and those among untreated individuals were 424 cells/microliter. The median HIV viral load of treated patients was 26,598 copies/ml, whereas that of untreated subjects was 10,848 copies/ml (Table 1).

Twenty-nine of the 38 (76%) samples analyzed provided molecular information for all four HIV genomic regions under study (PR, RT-POL, RT-CN and RT-RH). Three regions were sequenced for three samples (8%), two regions for five samples (13%) and one region for only one sample (3%). HIV-1 classification of the viral isolates based on this information revealed a complex pattern of HIV-1 subtypes and recombinant forms: 13 (34%) were subtype C; five (13%) were subtype B; the remaining 20 (53%) viruses had intersubtype recombinant structures. These were represented by ten CRF31_BC (26%), nine URF_BC (24%) and one URF_BCF (3%). All viruses had their pure or recombinant structure and recombination breakpoints (when applicable) confirmed by *bootscanning* analysis (data not shown).

Upon sequence analysis, 19 (50%) subjects had unclear electropherogram peaks, suggestive of infection by multiple subtypes/variants. These were further subject to clonal analysis of viral PCR products. However, clonal analyses did not show further evidence of multiple infections, and only a single variant type was found in each sample, confirming the original HIV subtype classification (data not shown).

**Figure 1:**
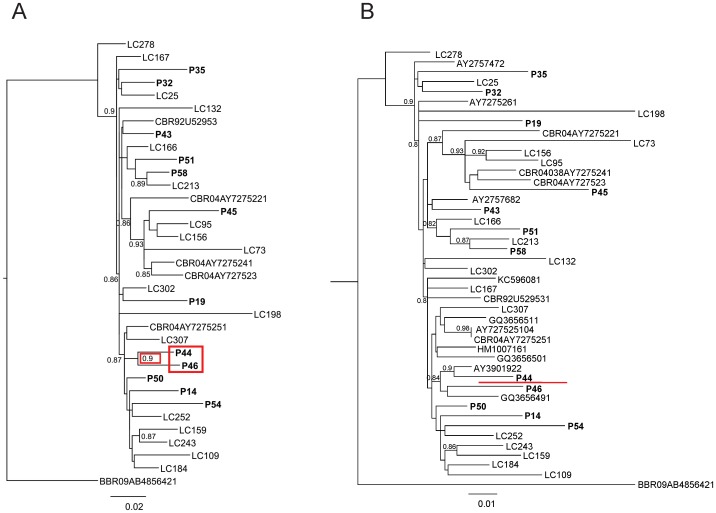
Phylogenetic analysis of HIV intraprison transmission clusters. ***A***, Maximum likelihood analysis between inmate (P) and local control (LC) HIV-1 subtype C sequences. P sequences generated in this study are shown in bold face. The clustering of inmate sequences P44 and P46 with high bootstrap support is boxed in red, suggesting a transmission link. In addition to LC sequences from Porto Alegre, reference HIV-1C sequences retrieved from GenBank are included. An HIV-1B reference sequence was used to root the tree. The aligned region corresponded to 730 bp. Only aLRT values greater than 0.85 are shown. ***B***, Same as in ***A***, except for the inclusion of ten additional sequences retrieved from the GenBank database which corresponded to the top-ten matched HIV sequences using the P44 and P46 sequences as query in a Blast analysis. The red line denotes the split between P44 and P46.

To estimate the degree of intraprison HIV transmission chains, viral strains were separated into subtype-specific groups and were analyzed phylogenetically for recent common ancestry. Strains belonging to each of the major subtype/CRF groups (subtype B, C or CRF31_BC) were aligned with LC sequences of the same subtype isolated from subjects followed up in a reference hospital of Porto Alegre (outside the prison). Whereas no two subtype B or CRF31_BC strains isolated from the prison grouped together, two subtype C strains (P44 and P46) did cluster with high aLRT support, suggesting that these strains were part of a transmission cluster, either through a direct link or by sharing a common ancestor (Figure 1A). To further assess the robustness of this suggestive intraprison cluster, both P44 and P46 sequences were blasted against the database, and the ten best hits were retrieved for additional phylogenetic inference. Of note, some of the best hits found in GenBank were common to both P44 and P46 sequences, providing an additional evidence of their relatedness. However, upon this new phylogenetic analysis, the cluster between P44 and P46 was split by two database sequences, ruling out the hypothesis of intraprison direct transmission (Figure 1B).

The genetic analysis of the HIV *pol* gene infecting the studied inmates enabled us to assess their patterns of antiretroviral drug resistance. Despite the fact that only seven (18%) subjects had reported previous/current treatment (Table 1), nine (24%) carried viruses with drug resistance mutations; five were treatment-experienced, while four were drug-naïve (Table 2). Of the latter, one virus carried the K103N major non- nucleoside reverse transcriptase inhibitor (NNRTI) mutation, one carried major protease inhibitor mutations, and two carried only C-terminal RT mutations of relevance to NRTI/NNRTI drug resistance (Table 2).

**Table 2:** Antiretroviral resistance mutations identified by direct sequencing and by clonal analysis.

SUBJECT	MUTATIONS				TREATMENT	ARV
	PR	RT	CN	RNase H		
P12	N/S	0	*G335D*	Q547K	No	X
P21	0	NNRTI: K103N	0	0	No	X
P23	0	NNRTI: K103N	0	0	Yes	N/A
P28	I54IV, L90M	0	0	0	No	X
P31	0	0	A371V	0	No	X
P33	0	0	A376S	0	Yes	N/A
P39	0	NNRTI: K103N	0	0	Yes	N/A
P46	*L10V*	NRTI: M184V; NNRTI: K103N, P225H	N348I	0	Yes	EFV Biovir
P54	0	NRTI: D67N, K70R,M184V,T215F,K219Q; NNRTI: A98G, K101P, K103N	*G335D*	Q509L	Yes	N/A

In italics, polymorphisms or secondary mutations.

X – not applicable.

0– no mutations.

N/A – not available.

N/S – not sequenced.

Of note, the HIV strain from patient P46 carried drug resistance mutations (Table 2) which were not observed in patient P44, further suggesting strengthening the lack of a direct HIV transmission event between these two subjects.

## Discussion

HIV prevalence within prisons has been consistently reported as significantly higher than in the respective general population both in developed and developing settings [5]. However, studies involving incarcerated subjects are poorly reported in low- and middle-income countries. In Brazil, studies conducted in the last decade reported HIV prevalence rates ranging from 5.7 to 14.5% in several cities of the São Paulo state, the most populated in the country [6], [36]–[38]. Our results are in agreement with the lower values of this range (6.6%), an estimate approximately 8-fold higher than the one reported to the general male population in the country [7]. A lower prevalence (2%), however, has been observed in the Rio de Janeiro state prison system [39]. Despite the fact that our survey screened nearly 40% of the entire incarcerated population of the unit, it may not represent the unit spatially, as only specific aisles (B and C) have been screened. The fact that our study population was not randomly selected is a limitation of our study, but random selection is operationally difficult in a prison population. On the other hand, those two aisles harbor all inmates already condemned, therefore representing a defined incarcerated population, to which further healthcare implementations can be effective. Moreover, the reasons underlying the reduction in the number of inmates with molecular HIV characterization, which included transfer to other prisons and liberation, have no plausible relationship with a risk of pertaining to potential transmission pairs or carrying drug resistance mutations. Therefore, we think those limitations did not influence the results presented herein.

A scenario of HIV-1 subtype C predominance, followed by its recombinants CRF31_BC and URF_BC was found in the incarcerated population studied here, which is in agreement with recent reports conducted in the city of Porto Alegre and in other areas of southern Brazil [16], [17], [21], [40]–[42]. These results suggest that the HIV molecular epidemics found inside this prison is not distinguishable from that of the local general population, a consequence of the intense flow between the prison and the outside population. In particular, the high rate of URF_BC found here may reflect multiple exposures to HIV of the population investigated. High-risk characteristics of the subjects under study, such as drug use (97%), sexual activity with commercial sex workers (26%) and tattooing inside the prison (37%) are likely to facilitate the occurrence and detection of these uncommon molecular findings.

In the present work, we did not find any evidence of intraprison HIV transmission. Despite one putative transmission pair of HIV sequences was initially evidenced (Fig. 1A), a more in-depth analysis with closely-related sequences from the GenBank database retrieved by Blast split the pair (Fig. 1B). Noteworthy, these sequences were derived from earlier studies conducted in the city of Porto Alegre (one characterized by our group [11] and the other by Passaes et al. [43]), further corroborating the idea that both inmates were infected while outside the prison. The lack of correlation between the HIV drug resistance mutation profiles of both inmates adds to this hypothesis. While the virus from P46 carried multiple mutations (Table 2), the one from P44 only harbored the G335D polymorphism in the HIV RT connection domain. Finally, a more detailed assessment of the incarceration history of the two inmates revealed that P46 had already been incarcerated for approximately nine years before our survey, and knew about his HIV^+^ status, while P44 was incarcerated 14 months before the survey, and only became aware of his HIV^+^ status during our screening. Altogether, such evidence strongly refutes the initial suggested cluster between these inmates, and overall no contribution of intraprison HIV transmission could be identified in our study. Yet there are only a few studies trying to address this issue in correctional facilities, a recent study conducted in central Brazil showed a total prevalence of 25% (4/16) among HIV-positive male inmates involved in intraprison viral clusters [9], a scenario distinct from ours. Despite that study included local controls from the same geographic area in the phylogenetic analysis like ours, no attempts to split the transmission clusters have been carried out with the inclusion of Blast-derived highly-related sequences, what could have rendered the authors’ hypothesis of linkage more robust.

The results of our study also point out to a high susceptibility of the study subjects to suboptimal antiretroviral therapy (ART). A high proportion of HIV strains carrying drug resistance mutations (24%) were observed, and almost half of the inmates infected with these strains (4/9) have reported no previous ART exposure. Even in the most conservative scenario, by not considering the recently characterized RT C-temrinal mutations), two out of 31 (6%) of untreated and four out of seven (57%) treated subjects carried primary resistance mutations. Most importantly, however, was that six out of the seven (86%) treated inmates were experiencing virological failure during our survey. In addition to the likely sub-notification of ARV drug exposure, we can also envisage deficient ARV supply, low adherence and irregular medical follow-up as drivers to those high rates of resistance and virological failure in this hard-to-reach population, as suggested by the high viral load and the limited number of subjects with undetectable viral load among the HIV-infected subjects we investigated.

In conclusion, our study contributes to the unveiling of the social health conditions the incarcerated population of Brazil are subject to. Public policy-makers should project into providing better access to adequate prevention measures, HIV screening at entry in prison and among already incarcerated inmates, sustainable access to ARV therapy and laboratorial monitoring, ARV adherence, medical care and better targeted STD prevention initiatives to people deprived of freedom in correctional facilities across the country to reduce intraprison HIV transmission.

## References

[1] Baral S, Sifakis F, Cleghorn F, Beyrer C (2007) Elevated risk for HIV infection among men who have sex with men in low- and middle-income countries 2000–2006: a systematic review. PLoS Med 4: e339. 18052602 10.1371/journal.pmed.0040339 PMC2100144

[2] Price MA, Rida W, Mwangome M, Mutua G, Middelkoop K, et al (2012) Identifying at-risk populations in Kenya and South Africa: HIV incidence in cohorts of men who report sex with men, sex workers, and youth. J Acquir Immune Defic Syndr 59: 185–193. 22227488 10.1097/QAI.0b013e31823d8693

[3] United Nations Office on Drugs and Crime & Joint United Nations Programme on HIV/AIDS (2009) WHO, UNODC, UNAIDS technical guide for countries to set targets for universal access to HIV prevention, treatment and care for injecting drug users. Geneva: World Health Organization.

[4] United Nations Office on Drugs and Crime & Joint United Nations Programme on HIV/AIDS (2009) Policy Brief: HIV testing and counseling in prison and other closed settings. Geneva: World Health Organization.

[5] Dolan K, Kite B, Black E, Aceijas C, Stimson GV (2007) HIV in prison in low-income and middle-income countries. Lancet Infect Dis 7: 32–41. 17182342 10.1016/S1473-3099(06)70685-5

[6] Coelho HC, Perdona GC, Neves FR, Passos AD (2007) HIV prevalence and risk factors in a Brazilian penitentiary. Cad Saude Publica 23: 2197–2204. 17700954 10.1590/s0102-311x2007000900027

[7] Ministério da Saúde do Brasil (2011) Boletim Epidemiológico Aids e DST In: DST/Aids e Hepatites Virais, editor. Brasília: MInistério da Saúde do Brasil.

[8] Burattini M, Massad E, Rozman M, Azevedo R, Carvalho H (2000) Correlation between HIV and HCV in Brazilian prisoners: evidence for parenteral transmission inside prison. Rev Saude Publica 34: 431–436. 11105105 10.1590/s0034-89102000000500001

[9] Cardoso LP, da Silveira AA, Francisco RB, da Guarda Reis MN, Stefani MM (2011) Molecular characteristics of HIV type 1 infection among prisoners from Central Western Brazil. AIDS Res Hum Retroviruses 27: 1349–1353. 21732793 10.1089/aid.2011.0153 PMC3227242

[10] Robertson DL, Anderson JP, Bradac JA, Carr JK, Foley B, et al (2000) HIV-1 nomenclature proposal. Science 288: 55–56. 10766634 10.1126/science.288.5463.55d

[11] Soares EA, Santos RP, Pellegrini JA, Sprinz E, Tanuri A, et al (2003) Epidemiologic and molecular characterization of human immunodeficiency virus type 1 in southern Brazil. J Acquir Immune Defic Syndr 34: 520–526. 14657764 10.1097/00126334-200312150-00012

[12] Soares EA, Martinez AM, Souza TM, Santos AF, Da Hora V, et al (2005) HIV-1 subtype C dissemination in southern Brazil. AIDS 19 Suppl 4S81–86. 16249660 10.1097/01.aids.0000191497.00928.e4

[13] Fontella R, Soares MA, Schrago CG (2008) On the origin of HIV-1 subtype C in South America. AIDS 22: 2001–2011. 18784462 10.1097/QAD.0b013e3283108f69

[14] Bello G, Passaes CP, Guimaraes ML, Lorete RS, Matos Almeida SE, et al (2008) Origin and evolutionary history of HIV-1 subtype C in Brazil. AIDS 22: 1993–2000. 18753928 10.1097/QAD.0b013e328315e0aa

[15] Bello G, Zanotto PM, Iamarino A, Graf T, Pinto AR, et al (2012) Phylogeographic analysis of HIV-1 subtype C dissemination in Southern Brazil. PLoS One 7: e35649. 22530062 10.1371/journal.pone.0035649 PMC3329557

[16] Bello G, Soares MA, Schrago CG (2011) The Use of Bioinformatics for Studying HIV Evolutionary and Epidemiological History in South America. AIDS Res Treat 2011: 154945. 22162803 10.1155/2011/154945 PMC3226295

[17] Silveira J, Santos AF, Martinez AM, Goes LR, Mendoza-Sassi R, et al (2012) Heterosexual transmission of human immunodeficiency virus type 1 subtype C in southern Brazil. J Clin Virol 54: 36–41. 22326760 10.1016/j.jcv.2012.01.017

[18] Santos AF, Lengruber RB, Soares EA, Jere A, Sprinz E, et al (2008) Conservation patterns of HIV-1 RT connection and RNase H domains: identification of new mutations in NRTI-treated patients. PLoS One 3: e1781. 18335052 10.1371/journal.pone.0001781 PMC2262134

[19] Santos AF, Silveira J, Muniz CP, Tornatore M, Goes LR, et al (2011) Primary HIV-1 drug resistance in the C-terminal domains of viral reverse transcriptase among drug-naive patients from Southern Brazil. J Clin Virol 52: 373–376. 21975076 10.1016/j.jcv.2011.09.005

[20] Soares EA, Makamche MF, Siqueira JD, Lumngwena E, Mbuagbaw J, et al (2010) Molecular diversity and polymerase gene genotypes of HIV-1 among treatment-naive Cameroonian subjects with advanced disease. J Clin Virol 48: 173–179. 20483657 10.1016/j.jcv.2010.04.008

[21] Santos AF, Schrago CG, Martinez AM, Mendoza-Sassi R, Silveira J, et al (2007) Epidemiologic and evolutionary trends of HIV-1 CRF31_BC-related strains in southern Brazil. J Acquir Immune Defic Syndr 45: 328–333. 17496564 10.1097/QAI.0b013e3180690d6a

[22] Hall TA (1999) BioEdit: a user-friendly biological sequence alignment editor and analysis program for Windows 95/98/NT. Nucl Acids Symp Ser 41: 95–98.

[23] Guindon S, Dufayard JF, Lefort V, Anisimova M, Hordijk W, et al (2010) New algorithms and methods to estimate maximum-likelihood phylogenies: assessing the performance of PhyML 3.0. Syst Biol 59: 307–321. 20525638 10.1093/sysbio/syq010

[24] Keane TM, Creevey CJ, Pentony MM, Naughton TJ, McLnerney JO (2006) Assessment of methods for amino acid matrix selection and their use on empirical data shows that ad hoc assumptions for choice of matrix are not justified. BMC Evol Biol 6: 29. 16563161 10.1186/1471-2148-6-29 PMC1435933

[25] Anisimova M, Gascuel O (2006) Approximate likelihood-ratio test for branches: A fast, accurate, and powerful alternative. Syst Biol 55: 539–552. 16785212 10.1080/10635150600755453

[26] Tamura K, Peterson D, Peterson N, Stecher G, Nei M, et al (2011) MEGA5: molecular evolutionary genetics analysis using maximum likelihood, evolutionary distance, and maximum parsimony methods. Mol Biol Evol 28: 2731–2739. 21546353 10.1093/molbev/msr121 PMC3203626

[27] Lole KS, Bollinger RC, Paranjape RS, Gadkari D, Kulkarni SS, et al (1999) Full-length human immunodeficiency virus type 1 genomes from subtype C-infected seroconverters in India, with evidence of intersubtype recombination. J Virol 73: 152–160. 9847317 10.1128/jvi.73.1.152-160.1999 PMC103818

[28] Johnson VA, Calvez V, Gunthard HF, Paredes R, Pillay D, et al (2011) 2011 update of the drug resistance mutations in HIV-1. Top Antivir Med 19: 156–164. 22156218 PMC6148877

[29] Delviks-Frankenberry KA, Nikolenko GN, Maldarelli F, Hase S, Takebe Y, et al (2009) Subtype-specific differences in the human immunodeficiency virus type 1 reverse transcriptase connection subdomain of CRF01_AE are associated with higher levels of resistance to 3′-azido-3′-deoxythymidine. J Virol 83: 8502–8513. 19553318 10.1128/JVI.00859-09 PMC2738196

[30] Delviks-Frankenberry KA, Lengruber RB, Santos AF, Silveira JM, Soares MA, et al. (2012) Connection subdomain mutations in HIV-1 subtype-C treatment-experienced patients enhance NRTI and NNRTI drug resistance. Virology. 10.1016/j.virol.2012.09.021 PMC3534945 23068886

[31] Brehm JH, Koontz D, Meteer JD, Pathak V, Sluis-Cremer N, et al (2007) Selection of mutations in the connection and RNase H domains of human immunodeficiency virus type 1 reverse transcriptase that increase resistance to 3′-azido-3′-dideoxythymidine. J Virol 81: 7852–7859. 17507476 10.1128/JVI.02203-06 PMC1951314

[32] Lengruber RB, Delviks-Frankenberry KA, Nikolenko GN, Baumann J, Santos AF, et al (2011) Phenotypic characterization of drug resistance-associated mutations in HIV-1 RT connection and RNase H domains and their correlation with thymidine analogue mutations. J Antimicrob Chemother 66: 702–708. 21393163 10.1093/jac/dkr005 PMC3058567

[33] Ehteshami M, Beilhartz GL, Scarth BJ, Tchesnokov EP, McCormick S, et al (2008) Connection domain mutations N348I and A360V in HIV-1 reverse transcriptase enhance resistance to 3′-azido-3′-deoxythymidine through both RNase H-dependent and -independent mechanisms. J Biol Chem 283: 22222–22232. 18547911 10.1074/jbc.M803521200 PMC2494928

[34] Tanuma J, Hachiya A, Ishigaki K, Gatanaga H, Lien TT, et al (2010) Impact of CRF01_AE-specific polymorphic mutations G335D and A371V in the connection subdomain of human immunodeficiency virus type 1 (HIV-1) reverse transcriptase (RT) on susceptibility to nucleoside RT inhibitors. Microbes Infect 12: 1170–1177. 20713171 10.1016/j.micinf.2010.08.003

[35] Dau B, Ayers D, Singer J, Harrigan PR, Brown S, et al (2010) Connection domain mutations in treatment-experienced patients in the OPTIMA trial. J Acquir Immune Defic Syndr 54: 160–166. 20130473 10.1097/QAI.0b013e3181cbd235

[36] Lopes F, Latorre MR, Campos Pignatari AC, Buchalla CM (2001) [HIV, HPV, and syphilis prevalence in a women’s penitentiary in the city of Sao Paulo, 1997–1998]. Cad Saude Publica 17: 1473–1480. 11784908 10.1590/s0102-311x2001000600018

[37] Strazza L, Azevedo RS, Carvalho HB, Massad E (2004) The vulnerability of Brazilian female prisoners to HIV infection. Braz J Med Biol Res 37: 771–776. 15107941 10.1590/s0100-879x2004000500020

[38] Guimaraes T, Granato CF, Varella D, Ferraz ML, Castelo A, et al (2001) High prevalence of hepatitis C infection in a Brazilian prison: identification of risk factors for infection. Braz J Infect Dis 5: 111–118. 11506773 10.1590/s1413-86702001000300002

[39] Sanchez A, Gerhardt G, Natal S, Capone D, Espinola A, et al (2005) Prevalence of pulmonary tuberculosis and comparative evaluation of screening strategies in a Brazilian prison. Int J Tuberc Lung Dis 9: 633–639. 15971390

[40] de Medeiros RM, Junqueira DM, Matte MC, Barcellos NT, Chies JA, et al (2011) Co-circulation HIV-1 subtypes B, C, and CRF31_BC in a drug-naive population from Southernmost Brazil: analysis of primary resistance mutations. J Med Virol 83: 1682–1688. 21837783 10.1002/jmv.22188

[41] Graf T, Passaes CP, Ferreira LG, Grisard EC, Morgado MG, et al (2011) HIV-1 genetic diversity and drug resistance among treatment naive patients from Southern Brazil: an association of HIV-1 subtypes with exposure categories. J Clin Virol 51: 186–191. 21622023 10.1016/j.jcv.2011.04.011

[42] Brigido LF, Nunes CC, Oliveira CM, Knoll RK, Ferreira JL, et al (2007) HIV type 1 subtype C and CB Pol recombinants prevail at the cities with the highest AIDS prevalence rate in Brazil. AIDS Res Hum Retroviruses 23: 1579–1586. 18160017 10.1089/aid.2007.0102

[43] Passaes CP, Guimaraes ML, Bello G, Morgado MG (2009) Near full-length genome characterization of HIV type 1 unique BC recombinant forms from Southern Brazil. AIDS Res Hum Retroviruses 25: 1339–1344. 19954300 10.1089/aid.2009.0167

